# Possible contamination of expressed breast milk by SARS‐CoV‐2

**DOI:** 10.1111/ped.14607

**Published:** 2021-06-27

**Authors:** Shinsuke Mizuno, Ken‐ichiro Kobayashi, Kenji Kubo, Nobuhiro Komiya

**Affiliations:** ^1^ Japanese Red Cross Society Wakayama Medical Center Wakayama Japan

**Keywords:** contamination, COVID‐19, educational intervention, expressed breast milk, mother‐child transmission

On 15 April 2020, a 3‐month‐old Japanese girl tested positive for severe acute respiratory coronavirus 2 (SARS‐CoV‐2) by real‐time reverse transcriptase‐polymerase chain reaction (RT‐PCR). She was born at 39 weeks of gestation and her birthweight was 3,309 g. There was no significant postnatal medical history. She had no siblings and no other notable family history. On 10 April 2020, her father tested positive for coronavirus disease 2019 (COVID‐19) by RT‐PCR. Reverse transcriptase‐polymerase chain reaction tests on nasopharyngeal and oropharyngeal swab specimens taken from the infant and her mother were tested repeatedly as close contact cases. On 10 April, the mother's RT‐PCR test was positive; she was admitted to other hospital on 11 April. Because the infant's first RT‐PCR test was negative, the mother left the infant with her aunt. Before her mother was admitted to other hospital, the infant was fed only breast milk. On 15 April, nasopharyngeal and oropharyngeal swab specimens from the infant tested positive. The infant was then admitted to our hospital on 16 April. The mother's breast milk was collected by either manual expression or breast pump at home on 11 April, and the result of the RT‐PCR test was positive. The threshold cycle of the expressed breast milk was 31.9. Later, on 14 April and after the mother was given appropriate advice on how to express breast milk by medical professionals, including disinfecting the hands with 70% ethanol, wiping the breasts with cleaning cotton and wearing gloves and a face mask, no SARS‐CoV‐2 was detected in expressed breast milk. The infant was fed with formula milk in the meanwhile to prevent possible transmission through expressed breast milk. The mother is 29 years old. She had a mild fever and taste disorders but no respiratory symptoms, and was in good general health. The infant had no fever and no respiratory symptoms on admission but developed transient mild respiratory symptoms while in hospital. Japan uses a test‐based strategy to control the virus. After resolution of symptoms, individual samples are checked by RT‐PCR; negative results from at least two consecutive respiratory samples collected more than 24 h apart are required before an individual is regarded as virus free. The mother and infant fulfilled both of these requirements and were discharged from hospital on 5 May (Fig. 1).

**Fig. 1:**
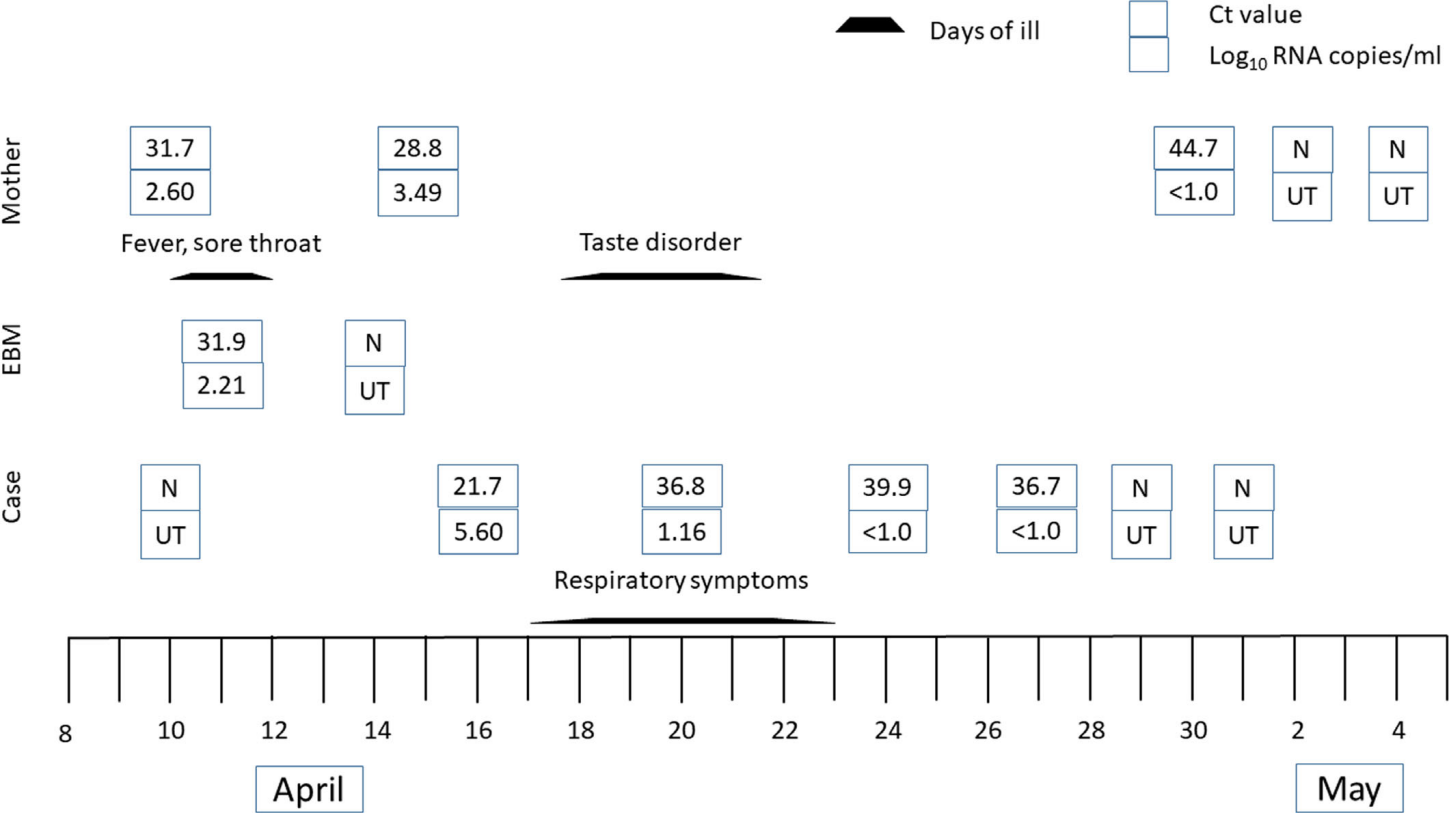
Clinical information regarding the case and the mother. Isolation of SARS‐CoV‐2 in nasopharyngeal swab, oropharyngeal swab and expressed breast milk. Ct, cycle threshold; N, negative for SARS‐CoV‐2 RNA by RT‐PCR; UT, untypable.

Since December 2019, novel COVID‐19, which is caused by SARS‐CoV‐2, has spread rapidly around the world.^1^ There is little high‐quality evidence relating to transmission of SARS‐CoV‐2 to infants. It is unclear whether the virus can be transmitted through breast milk because very few breast milk samples have been tested.^2^, ^3^ Investigation of past reports of breast milk from mothers with COVID‐19 indicates that only a few samples were positive results for SARS‐CoV‐2 by RT‐PCR test.^4^ In this report, samples of breast milk were taken by patients themselves with no education about appropriate protective measures. So further study is needed to determine that breast milk can be transmissible even if proper protective measures are taken. Guidance issued by the Centers for Disease Control and Prevention (CDC) recommends that mothers should receive support to enable them to continue to breastfeed. By contrast, the Japanese Society of Obstetrics and Gynecology recommends the use of formula milk. Expressed breast milk may be contaminated through inappropriate handling.^5^ Breastfeeding has both short‐ and long‐term benefits for the mother and infant. Human breast milk contains factors that protect against infectious disease and promote immune system development. And temporarily interruption of breastfeeding may cause mastitis, decreasing milk secretion, and mental stress on the mother. Here, we describe the clinical course of an infant who was fed breast milk from a mother with COVID‐19. We also describe the results of tests carried out on the expressed breast milk.

We detected SARS‐CoV‐2 RNA in expressed breast milk from the mother with COVID‐19. Testing of consecutive samples of expressed breast milk over a short time interval yielded varying results. The estimated viral load in expressed breast milk samples obtained with inadequate disinfection of hands and breast, inappropriate wearing of the personal protective equipment was quite high; however, after educational intervention and use of personal protective equipment, no virus was detected in expressed milk samples collected at later time points. During this time, the mother's viral load was considered to be high. It is possible to have a false‐positive result due to contamination while processing the laboratory samples. Additionally, there is a slim possibility of transmitting virus via direct contact from mother's skin to baby. For this case, therefore, the test result should better be translated as false‐positive as the baby's polymerase chain reaction (PCR) was negative and that of the mother's breast milk was positive. It is still unclear whether the infant was infected by breast milk or other modes of transmission but these findings indicate that it is possible to contaminate expressed breast milk when expression is carried out without appropriate measures are not taken. Effective guidance and education regarding breastmilk expression is therefore required. The study protocol was approved by the Institutional Review Board of the Japanese Red Cross Wakayama Medical Center (no. 816).

## Disclosure

The authors declare no conflicts of interest.

## Author contribution

Dr Shinsuke Mizuno conceptualized and designed the study, drafted the initial manuscript, and reviewed and revised the manuscript. Dr Ken‐ichiro Kobayashi, Kenji Kubo, and Nobuhiro Komiya designed the data‐collection instruments, coordinated and supervised the data collection, and critically reviewed the manuscript. All authors approved the final manuscript as submitted and agree to be accountable for all aspects of the work.
